# Using Supervised Learning Methods for Gene Selection in RNA-Seq Case-Control Studies

**DOI:** 10.3389/fgene.2018.00297

**Published:** 2018-08-03

**Authors:** Stephane Wenric, Ruhollah Shemirani

**Affiliations:** ^1^Laboratory of Human Genetics, GIGA-Research, University of Liège, Liège, Belgium; ^2^Department of Genetics and Genomic Sciences, The Charles Bronfman Institute for Personalized Medicine, Icahn School of Medicine at Mount Sinai Hospital, New York, NY, United States; ^3^Department of Computer Science, Information Sciences Institute, University of Southern California, Marina del Rey, CA, United States

**Keywords:** RNA-Seq, supervised learning, random forests, variational autoencoders, gene selection, feature selection, transcriptomics, gene expression

## Abstract

Whole transcriptome studies typically yield large amounts of data, with expression values for all genes or transcripts of the genome. The search for genes of interest in a particular study setting can thus be a daunting task, usually relying on automated computational methods. Moreover, most biological questions imply that such a search should be performed in a multivariate setting, to take into account the inter-genes relationships. Differential expression analysis commonly yields large lists of genes deemed significant, even after adjustment for multiple testing, making the subsequent study possibilities extensive. Here, we explore the use of supervised learning methods to rank large ensembles of genes defined by their expression values measured with RNA-Seq in a typical 2 classes sample set. First, we use one of the variable importance measures generated by the random forests classification algorithm as a metric to rank genes. Second, we define the EPS (extreme pseudo-samples) pipeline, making use of VAEs (Variational Autoencoders) and regressors to extract a ranking of genes while leveraging the feature space of both virtual and comparable samples. We show that, on 12 cancer RNA-Seq data sets ranging from 323 to 1,210 samples, using either a random forests-based gene selection method or the EPS pipeline outperforms differential expression analysis for 9 and 8 out of the 12 datasets respectively, in terms of identifying subsets of genes associated with survival. These results demonstrate the potential of supervised learning-based gene selection methods in RNA-Seq studies and highlight the need to use such multivariate gene selection methods alongside the widely used differential expression analysis.

## Introduction

Transcriptomics studies making use of RNA-Seq usually produce large amounts of data, namely one expression value for each gene or transcript of each sample assessed (Mortazavi et al., 2008; Wang et al., 2009).

Searching for genes of interest or prioritizing genes in the context of case-control studies related to diseases or other experimental conditions constitutes an important task ascribed to RNA-Seq experiments (Trapnell et al., 2009; Garber et al., 2011; Love et al., 2014; Wenric et al., 2017).

Current methods often make use of differential expression analysis, to select genes of interest and assign them a *p*-value related to a statistical test assessing changes in expression between different conditions.

Most commonly used software packages performing differential expression analysis make use of the negative binomial distribution to model read counts for each gene. This distribution, which is an extension of the Poisson distribution, has two parameters: the mean and the dispersion, which allows modeling of more general mean–variance relationships than Poisson. The dispersion parameter allows to take into account the biological variability arising in RNA-Seq data (Love et al., 2014; Huang et al., 2015).

However, even though software packages like DESeq2 model relationships between genes by assuming that genes of similar average expression have a similar dispersion, the statistical test conducted to assess significance is a univariate test performed independently for each gene. Albeit providing particularly useful and usually accurate information regarding disruptions of gene expression between conditions, these methods thus do not take into account the potential correlation and concordant or discordant effect between groups of genes. However, such gene-gene interactions are present in most tissues and conditions and they are known to play key roles in said conditions, with groups of genes which might have a significant effect as a group but not when each gene is considered independently (Kanehisa and Goto, 2000; Joshi-Tope et al., 2005; Phillips, 2008; Vidal et al., 2011).

Here, we explore the use of multivariate classifiers to rank genes in a case-control RNA-Seq experiment. Namely, we're using the permutation importance of the random forests classifier to rank genes, and a newly developed method Extreme Pseudo-Samples (EPS) making use of Variational Autoencoders.

Machine learning methods are progressively being applied to problems arising in genomics related fields and the idea of using importance measures generated by the random forests algorithm to extract a ranking of features has already been explored with several different data sets, although, to our knowledge, this has never been done with RNA-Seq data sets (Duro et al., 2012; Anaissi et al., 2013; Yao et al., 2015; Frères et al., 2016; Schrider and Kern, 2018).

Aside from random forests, we also introduce a technique called EPS allowing to create case and control pseudo-samples lying on the two extremes of the sample space. This method uses Variational Autoencoders (VAE; Kingma and Welling, 2013) to create new pseudo-samples that are not present in the original datasets but closely imitate their statistical properties, in that they share the properties of independent and identically distributed samples from the same distribution as the real data.

The idea of using autoencoders to classify and examine genomics datasets is not new (Tan et al., 2014). However, VAEs differ from other autoencoders in that they can create a meaningful latent representation space where one can choose a new vector in the latent space and create a valid, previously unseen sample in real space that closely follows the real samples (the aforementioned pseudo-samples).

Additionally, although autoencoders have been used as an auxiliary tool in the classification of existing datasets, no attempt has been made to extract the knowledge learnt by the autoencoders in this process to trace the analysis and results back to the actual gene expression values and their relationships. Here, we suggest a way to make use of that information (Tan et al., 2014).

In this work, we focus on the use of supervised learning algorithms solely to extract gene rankings, and not to actually perform samples classification.

## Materials and methods

### Data sets

Several data sets from the TCGA database have been selected to validate both methods (Weinstein et al., 2013).

Only the data sets containing 30 healthy samples (denoted as “Solid Tissue Normal” in the TCGA database) or more have been selected. All read counts produced by HTSeq as well as the clinical data have been downloaded with the TCGABiolinks R/Bioconductor package (Colaprico et al., 2015).

The data sets selected are summarized in Table 1.

**Table 1:** TCGA data sets used in this study.

**Name**	**Cancer type**	***N* (tumors)**	***n* (healthy)**	**Median age**	**Age range**
TCGA-BRCA	Breast invasive carcinoma	1,097	113	59.07	26-90
TCGA-LUAD	Lung adenocarcinoma	582	59	66.88	33-88
TCGA-UCEC	Uterine Corpus endometrial carcinoma	559	35	64.24	31-90
TCGA-KIRC	Kidney renal clear cell carcinoma	535	72	61.16	26-90
TCGA-HNSC	Head and neck squamous cell carcinoma	528	44	61.14	20-90
TCGA-THCA	Thyroid carcinoma	507	58	46.92	15-89
TCGA-LUSC	Lung squamous cell carcinoma	504	49	68.66	39-90
TCGA-PRAD	Prostate adenocarcinoma	498	52	61.99	42-78
TCGA-COAD	Colon adenocarcinoma	460	41	68.88	31-90
TCGA-STAD	Stomach adenocarcinoma	443	32	67.56	30-90
TCGA-LIHC	Liver hepatocellular carcinoma	377	50	61.53	16-88
TCGA-KIRP	Kidney renal papillary cell carcinoma	291	32	62.03	28-88

### Methodology

**Figure 1:**
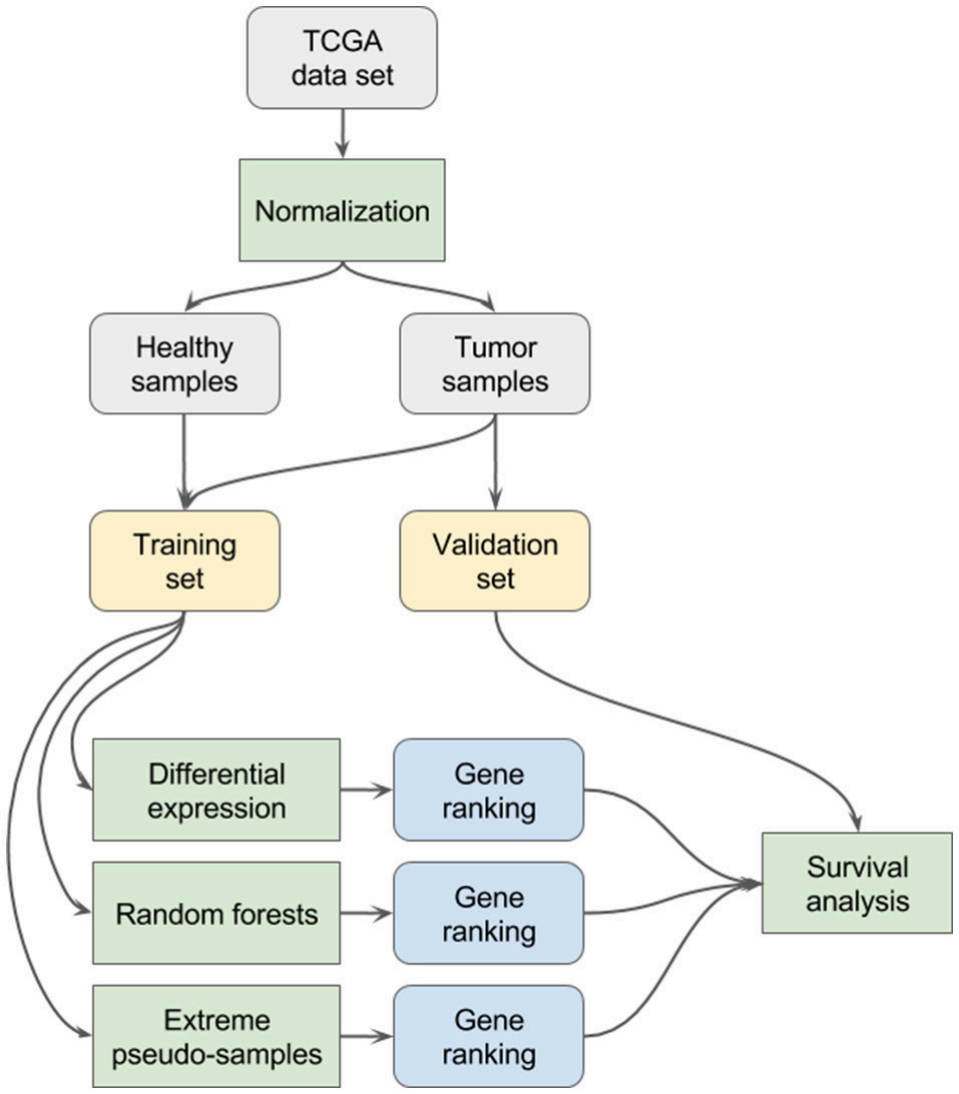
Study design: A diagram describing the methodology.

For each data set, the methodology illustrated in Figure 1 has been applied:

- All samples are normalized with the DESeq2 software package, using the default workflow parameters and commands suited for files generated by the *htseq-count* tool (namely the following R functions: *DESeqDataSetFromHTSeqCount, estimateSizeFactors, counts* with the *normalized* argument set to *TRUE*) as outlined in the reference manual of DESeq2 (Love et al., 2014).
- The samples are split into a training set and a validation set. The training set contains all the healthy samples of the original data set (*n*) and the same number of tumor samples as healthy samples (*n*). The validation set contains the remaining tumor samples (*N – n*).
- Differential expression analysis is performed on the training set with the DESeq2 software package, using default parameters and options. A ranking of genes, based on their adjusted *p*-value relative to the differential expression test, is obtained.
- A random forests classifier is built on the training set with the ranger R package, using 100,000 trees and a value for the *m*_*try*_ parameter of 236 (equal to the square root of the total number of features; Wright and Ziegler, 2015). A ranking of genes based on their permutation importance values is obtained (the permutation importance is computed by randomly permuting the values of the feature of interest and measuring the resulting increase in error).
- The EPS method (see section Extreme Pseudo-Sampling) is applied on the training set(s) to extract a ranking of genes.
- Let *RF* denote the random forests based gene ranking, *DE* the differential expression based gene ranking and *EPS* the extreme pseudo-samples based gene ranking. *RF*_*i*_ denotes the *i*-th gene of the random forests based gene ranking. Similarly, *DE*_*i*_ denotes the *i*-th gene of the differential expression based gene ranking and *EPS*_*i*_ denotes the *i*-th gene of the EPS based gene ranking.
- For both rankings, 20 gene signatures are generated, including an incremental number of genes. Let *sigRF*_*i*_ denote the *i*-th gene signature based on the random forests ranking, *sigDE*_*i*_ denote the *i*-th gene signature based on the differential expression ranking and *sigEPS*_*i*_ the *i*-th gene signature based on the EPS ranking. The signatures are formally defined as:
  ◦ *sigRF*_*i*_ = {*RF*_1_, …, *RF*_*i*_}, for *i* = 1, …, 20
  ◦ *sigDE*_*i*_ = {*DE*_1_, …, *DE*_*i*_}, for *i* = 1, …, 20
  ◦ *sigEPS*_*i*_ = {*EPS*_1_, …, *EPS*_*i*_}, for *i* = 1, …, 20
- For each signature,
  ◦ A Cox proportional hazard model was built using all genes of the signature
  ◦ The samples of the validation set were split into two groups (higher and lower survival), based on the median of the Cox proportional hazard model.
  ◦ A log-rank test was performed to compare the survival of the two groups.
- For *i* = {1, …, 20}, the *p*-value of the log-rank tests obtained with *sigDE*_*i*_, *sigRF*_*i*_, *sigEPS*_*i*_ are compared.

For each data set, correlation coefficients have been computed between the expression values of the 50% most expressed genes; a hierarchical clustering of the 50% most expressed genes was performed, to assess if multicollinearity played a role in the performance of the RF based method (multicollinearity denotes the presence of non-independent features such that the relationship between each of these features and the model output is influenced by the relationships between the non-independent features). A hierarchical clustering of all samples was also performed, with the 50% most expressed genes. Enrichment analysis was performed on gene lists from both methods.

The correlation coefficient between each top-ranked gene from both list and the 50% most expressed genes has been computed for each data set.

Globally, the correlation between the overall survival at 5 years of all cancer types, and the performance of the presented methods was computed.

For each gene ranking obtained for all the data sets, a gene set enrichment analysis has been performed using the *ConsensusPathDB* online tool (Kamburov et al., 2012).

### Extreme Pseudo-sampling

It is worth noting that, in most data sets considered in this study, the samples from both classes reside in a high dimensional space and are tightly coordinated together, such that a linear classifier cannot separate them at all. The low count of normal samples compared to the total sum of samples also contributes to the failure of linear classifiers; which tend to receive bias from such unbalance of class membership statistics.

We decided to use a dimensionality reduction technique in order to both address the *curse of dimensionality* and find a representation in which these samples lay in a linearly-separable subspace.

Autoencoders have shown to be able to create such latent representations better than their linear counterparts such as PCA (Tan et al., 2014; Danaee et al., 2017). However, such representations do not provide us with useful, actionable knowledge about genes due mainly to their non-linear activation functions.

Moreover, Normal Autoencoders are not generative, i.e., while it is possible to come up with useful latent representations for classification purposes, one cannot generate new samples similar to the real samples by slightly modifying their latent representation values and feeding the result into the decoder network.

A new type of Autoencoder, called the Variational Autoencoder, however, can succeed in this task (Kingma and Welling, 2013). VAEs are fundamentally different from other AEs in that they are generative models:

Each point *x* in real space will be associated with distribution *P(z|x)*. For the purpose of this methodology, we assumed this distribution to be normal. Getting latent representation *z*_1_ from sample *x*_1_, thus, would be equal to drawing a sample from distribution *N(*μ_1_, σ_1_*)*, where μ_1_, σ_1_ are learned from the training data.

The training VAE comprises 9 layers, having 30,000, 15,000, 10,000, 2,000, 500, 2,000, 10,000, 15,000, 30,000 perceptrons, respectively. The training process of these layers requires fine-tuning approximately 5 billion parameters. Given that the performance of this fine-tuning process increases with the number of samples, in addition to the training set extracted from the studied TCGA dataset, a random selection of samples from the 11 other training sets is used in the VAE training process.

After the training step, each dataset *D*_*c*_ is transformed to its latent representation *L*_*c*_. Said latent representation allows to linearly separate the normal samples from cancerous ones with almost 100% accuracy for both testing and training datasets. Considering the linear separator, let us denote the furthest populated areas on both sides of the separator, called *N*_*c*_ for the normal side of the linear separator and *C*_*c*_ for the cancerous side. If we consider a point *z*_*n*_ in one of these areas, we know it has been randomly drawn from distribution *N(*μ_*n*_, σ_*n*_*)*.

While selecting *z*_*n*_ is a random process, once a *z*_*n*_ has been drawn from any of the distributions, reconstructing ẋ_*n*_ ≈ *x*_*n*_ from *z*_*n*_ is a deterministic process done by the decoder. However, every point in the close proximity of *z*_*n*_ can be drawn from the same distribution. Due to the deterministic features of the decoder, each of these points would end up generating a different ẋ_*n*_. Although different, every possible ẋ_*n*_ should resemble the original *x*_*n*_ closely and should also follow the general statistical characteristics of all *x*'s in the dataset.

We then drew 400 random points in areas *N*_*c*_ *and C*_*c*_ of the latent space *L*_*c*_, on both sides of the linear separator and generated new “virtual” or “pseudo” samples of both cancerous and normal classes, a process that we call Extreme Pseudo Sampling (EPS). The amount of random points drawn (400) was chosen using cross validation on the training data. It was the smallest number of samples that ended up in a successful regression process.

While real samples cannot be divided using a linear separator and suffer from unbalance of class member counts; we were able to generate new pseudo samples that can be divided linearly in real space due to their exaggerated cancerous/normal features. These samples also are of equal count. The later trait enables the dividing regression lines to be less biased toward a specific class. Thus, said regression lines maintain the same distance from both classes.

Finally, since all sample features have been normalized in the process, weight coefficients in the line formula can be translated into importance factors for classifying extreme pseudo samples. The larger a coefficient, the more important its related feature is in determining class membership. Thereby, we are able to extract an importance ranking for all genes, in each data set.

The R and Python scripts used to perform the aforementioned analyses are available online: https://github.com/stephwen/ML_RNA-Seq & https://github.com/roohy/Extreme-Pseudo-Sampler

### Performance and stability measures

Both the random forests-based method and the EPS method are non-deterministic and benefit inherently from large sample sizes.

To assess the stability of the gene rankings produced by these 2 methods and the effect of smaller sample sizes, we employed the following two approaches:

First, we tested the stability of the gene rankings by performing the complete methodology described in section Methodology, on each TCGA dataset, 10 times. Given the focus on the highest ranked genes, we calculated, for each dataset, the number of genes in common amongst the top 20 genes across the 10 iterations. We also computed the average and standard deviation of the ranking of each gene reported in the initial run of the methodology, across these 10 iterations, for each dataset.

Second, we performed the methodology described in section Methodology, by using only a random selection of 20 percent of all samples (with a minimum of 20 healthy samples for the smallest datasets). We then compared the performances of the two supervised learning based methods with DESeq2, as described in section Methodology.

To further assess the benefit of supervised learning methods over deterministic univariate gene selection methods, we extracted a ranking of genes for each data set based on the magnitude of the absolute fold-change of each gene. The survival-centered methodology described in section Methodology has been applied to the fold-change based gene ranking, to obtain 20 *p*-values, which have then been compared to the log-rank *p*-values obtained with the three other methodologies.

## Results

For each data set, 60 log-rank tests have been performed on the validation set, using gene signatures *sigDE*_*i*_, *sigRF*_*i*_, and *sigEPS*_*i*_ with *i* = *{1, 2, …, 20}* which contain from 1 to 20 genes out of the gene ranking derived from differential expression analysis, the gene ranking derived from the random forests classifier, and the gene ranking derived from the EPS method respectively. The *p*-values of these tests have been compared two by two.

Table 2 summarizes the results and shows the number of gene signatures where the random forests-based gene ranking outperforms the differential expression-based gene ranking and where the Extreme-Pseudo Sampling method outperforms the differential expression-based gene ranking.

**Table 2:** Performance comparison of survival gene signatures: The random forests column denotes the number of random forests-based signatures having a lower log-rank *p*-value than their corresponding differential expression-based signatures.

**Name**	**Cancer type**	**Random forests**	**Extreme pseudo-samples**
TCGA-BRCA	Breast invasive carcinoma	5	19
TCGA-LUAD	Lung adenocarcinoma	14	14
TCGA-UCEC	Uterine Corpus endometrial carcinoma	16	9
TCGA-KIRC	Kidney renal clear cell carcinoma	13	10
TCGA-HNSC	Head and neck squamous cell carcinoma	14	15
TCGA-THCA	Thyroid carcinoma	15	15
TCGA-LUSC	Lung squamous cell carcinoma	5	0
TCGA-PRAD	Prostate adenocarcinoma	12	19
TCGA-COAD	Colon adenocarcinoma	11	18
TCGA-STAD	Stomach adenocarcinoma	13	19
TCGA-LIHC	Liver hepatocellular carcinoma	19	8
TCGA-KIRP	Kidney renal papillary cell carcinoma	10	19

*The extreme pseudo-samples column denotes the number of extreme pseudo-samples-based signatures having a lower log-rank p-value than their corresponding differential expression-based signatures. The 3 colors (green, yellow, red) refer to cases where the proposed methods have a higher number, the same number, and a lower number of best-performing gene signatures than DESeq2, respectively*.

For 9 out of the 12 data sets analyzed (lung adenocarcinoma, uterine corpus endometrial carcinoma, kidney renal clear cell carcinoma, head, and neck squamous cell carcinoma, thyroid carcinoma, prostate adenocarcinoma, colon adenocarcinoma, stomach adenocarcinoma, liver hepatocellular carcinoma), the random forests-based gene ranking outperforms the differential expression-based gene ranking in terms of identifying subsets of genes associated with survival. For 8 out of the 12 datasets (breast invasive carcinoma, lung adenocarcinoma, head, and neck squamous cell carcinoma, thyroid carcinoma, prostate adenocarcinoma, colon adenocarcinoma, stomach adenocarcinoma, kidney renal papillary cell carcinoma), the EPS-based gene ranking outperforms the differential expression-based gene ranking. For one data set (kidney renal papillary cell carcinoma), both the DESEq2 and the random forests-based gene rankings share the same number of best performing signatures. For one data set (kidney renal clear cell carcinoma), both the DESEq2 and the EPS-based gene rankings share the same number of best performing signatures. For 2 out of the 12 data sets (breast invasive carcinoma, lung squamous cell carcinoma), the differential expression-based gene ranking outperforms the random forests-based gene ranking. For 3 out of the 12 data sets (uterine corpus endometrial carcinoma, lung squamous cell carcinoma, liver hepatocellular carcinoma), the differential expression-based gene ranking outperforms the EPS-based gene ranking.

**Figure 2:**
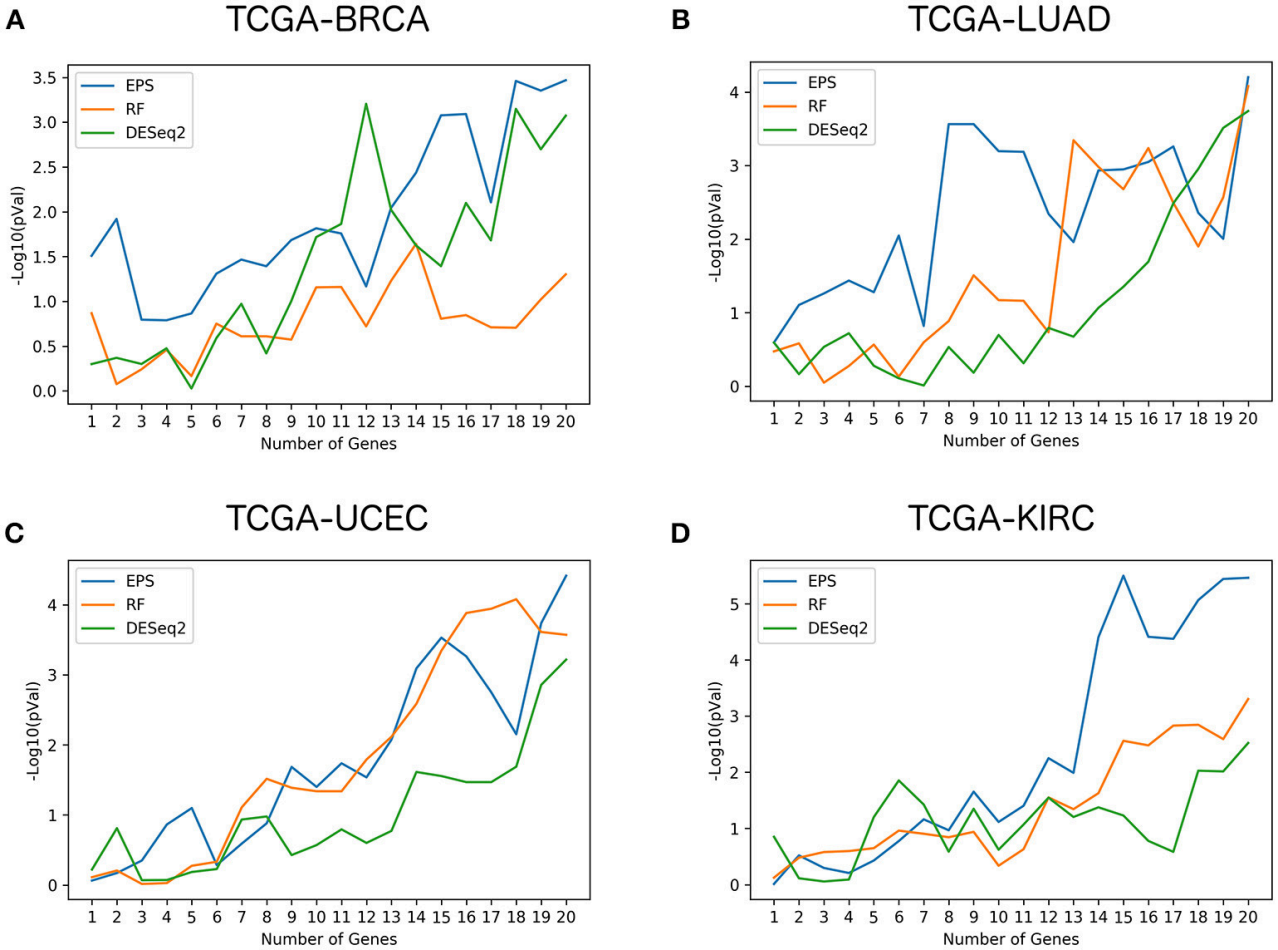
Performance comparison of survival gene signatures: Evolution of the log-rank p-values obtained with survival gene signatures comprising incremental number of genes, for the 3 methods compared and the 4 largest TCGA datasets.

Figure 2 shows the log-rank *p*-values for the 3 different methods (DESeq2, random forests, EPS) and their respective gene signatures ranging from 1 to 20 genes, for the 4 largest data sets (TCGA-BRCA, TCGA-LUAD, TCGA-UCEC, TCGA-KIRC). Similar figures for the 8 other data sets are available as Supplementary Figure 1. The log-rank *p*-values for the 20 gene signatures related to the 3 rankings for each dataset and the genome wide ranking of genes based on the permutation importance computed by the random forests classifier and on the EPS method can be found in Supplementary Tables 1, 2, respectively.

No significant difference in the average absolute correlation coefficient obtained between the 50% most expressed genes was found between the different cohorts whose DE based signatures performed better than the RF and EPS signatures and the cohorts whose RF or EPS based signatures performed better than the DE ones. No significant difference in terms of the number of clusters of samples obtained with a hierarchical clustering with the 50% most expressed genes when using a constant height cutoff value of *h* = 2^*^10^6^ was found between the different cohorts whose DE based signatures performed better than the RF and EPS signatures and the cohorts whose RF or EPS based signatures performed better than the DE ones. No significant difference in terms of the number of clusters of genes obtained with a hierarchical clustering with the 50% most expressed genes when using a constant height cutoff value of *h* = 10^5^ was found either. No significant difference was found between the correlation between the top-ranked genes selected with both methods and the 50% most expressed genes. No correlation was found between the overall survival at 5 years of the different cancer types and the performance of either method (measured as the ratio of n/20 top-performing signatures). There is, however, a loose correlation (Pearson correlation coefficient: 0.627, *p*-value: 0.029) between the number of best-performing DE based signatures among the 20 signatures of each data set and the number of differentially expressed genes (adjusted *p*-value < 0.05) in each data set. Correlation coefficients and numbers of clusters are present, for all data sets, in Supplementary Table 3.

A gene set enrichment analysis performed on the top ranked gene obtained via the random forests-based method and the EPS method yielded several cancer-related enriched pathways, as shown in Table 3.

**Table 3:** Results of a pathway-based gene set enrichment analysis performed on the top 20 ranked genes obtained through the supervised learning methods.

**Name**	**Cancer type**	**Gene ranking**	**Enriched pathway**	***P*-value**	**Source**
TCGA-BRCA	Breast invasive carcinoma	EPS	Signaling by PTK6 (Goel and Lukong, 2015)	0.00176	Reactome
TCGA-UCEC	Uterine Corpus endometrial carcinoma	RF	Oncostatin_M (Zhu et al., 2015; Junk et al., 2017)	0.000876	NetPath
		EPS	IGF1 (Baserga et al., 2003; Bruchim et al., 2014; Cao et al., 2015; Dai et al., 2016)	0.000622	PID
TCGA-HNSC	Head and neck squamous cell carcinoma	RF	PPAR signaling pathway (Michalik et al., 2004)	0.00278	Wikipathways
		EPS	AURKA (Chou et al., 2012)	0.00198	Reactome
TCGA-LUSC	Lung squamous cell carcinoma	EPS	IGF1 Integrated lung cancer pathway (Brabender et al., 2001)	0.000406 0.000724	PID Wikipathways
TCGA-PRAD	Prostate adenocarcinoma	EPS	IGF1 AURKA	0.000545 0.00311	PID Reactome
TCGA-COAD	Colon adenocarcinoma	RF	Mitochondrial Beta-Oxidation of Long Chain Saturated Fatty Acids (Wen et al., 2017) Liver steatosis (Sanna et al., 2016)	3.6e-05 0.000105	SMPDB Wikipathways
TCGA-LIHC	Liver hepatocellular carcinoma	RF	Angiogenesis (Muto et al., 2015)	0.00168	Wikipathways

Contrary to DESeq2, both the random forests-based method and the EPS method are non-deterministic. Therefore, the stability of the rankings obtained through these methods has been assessed. Through 10 iterations of the complete methodology, a distribution of the ranking of each gene has been obtained. The average and standard deviation of the gene ranking obtained for each of the 12 datasets and the 2 supervised learning methods are shown in Supplementary Table 1.

As expected, given the random parameters involved in these 2 supervised learning methods, most gene rankings vary across the different iterations of the methodology, with a lower variance for the best ranked genes. It should however be noted that, for the EPS method, a dataset has the same top-ranked genes across all iterations (TCGA-LUSC), while other datasets have a highly similar order (TCGA-HNSC, TCGA-LIHC). For the random forests-based method, in a select few datasets (TCGA-BRCA, TCGA-THCA, TCGA-COAD), the top-ranked gene systematically ends up at the first rank across the different iterations. We did not observe a correlation between the sample size of each dataset and the variance of the gene rankings. Despite these variations, there is a majority of genes in common amongst the top ranked genes, across 10 iterations. When using the random forests-based method, the average number of genes in common amongst the top 20, across 10 iterations varies from 15.5 (TCGA-BRCA) to 18.51 (TCGA-HNSC). When using the EPS method, the average number of genes in common amongst the top 20, across 10 iterations varies from 10.44 (TCGA-KIRC) to 20 (TCGA-HNSC, TCGA-LIHC, TCGA-LUSC). The average number of genes in common amongst the top 20, for all datasets and the 2 proposed methods, are shown in Supplementary Table 4.

Additionally, a fold-change magnitude-based gene selection method has been tested, yielding overall poorer results than the 2 proposed methods (see Supplementary Table 5).

When ran on a random selection of 20 percent of the initial samples, the number of datasets in which the random forests-based approach performs worse than DESeq2 grows from 2 to 5 datasets. For the EPS model, this grows from 3 to 4. Both methods still outperform DESeq2 in terms of finding survival associated gene signatures in more than 50% of the datasets (see Supplementary Table 6).

## Discussion

Highlighting genes of interest has always been a part of transcriptomics studies and the advent of RNA sequencing technologies has but further emphasized this endeavor. Traditionally, genes of interest, in case-control studies where one had access to their expression values, were genes where said expression varied greatly from one class to the other. This definition has led to the development of numerous methods making use of diverse statistical models and tests, achieving impressive results in a lot of different use cases. However, these methods often implicitly neglected the importance of gene-gene relationships, by only looking at univariate changes.

Here, we propose a paradigm shift, by directing the search for genes of interest toward the use of machine learning methods originally conceived to predict the membership of a sample in a class, as these methods intrinsically model the inter-variable relationships (i.e., the previously overlooked gene-gene links).

An obvious kind of data sets which should theoretically benefit from this are cancers, as these pathologies are known to involve several genes in a multistep process, with different mechanisms implicating intricate relationships between said genes (Yates and Campbell, 2012; Vogelstein et al., 2013).

By using 12 data sets containing samples of various cancers, we have shown that supervised classification algorithms could be used to extract a meaningful ranking of genes. Namely, the permutation importance (also known as Mean Decrease in Accuracy) generated by the random forests algorithm and the weights coefficients used in the EPS provided a ranking of genes which outperformed classical methods in most data sets.

The permutation importance is not the only variable importance generated by the random forests classifier, as the Gini importance (or Mean Decrease in Impurity) is also available. However, using the Gini importance to classify the genes of these data sets yielded slightly worse results than the results obtained with the permutation importance. Using a combination of both variable importances, as in Frères et al. (2016), also produced worse results than when using the permutation importance alone.

Given the fact that neither the random forests-based gene ranking nor the EPS based one outperformed the differential expression based one for all of the 12 data sets, one might wonder if using both a supervised learning-based gene selection technique in conjunction with differential expression would not yield better results. However, using the supervised learning-based gene selection method after the differential expression one (i.e., using only the genes with a significant differential expression adjusted *p*-value as input features of the random forests classifier or the EPS method) also produced worse results than when using the random forests gene ranking or the EPS gene ranking alone.

Using survival analysis as a way to validate gene lists coming from cancer data sets whose average survival differs greatly might spark questions, however there does not seem to be a link between the overall survival (OS) of these cancers and the performance of the proposed methods. Survival information constitutes a quantifiable and relatively easily available information for different data sets. However, using the presumed relationship between the expression values of a gene and the survival of a patient as a proxy for the role of said gene in the selected disease relies on a strong hypothesis whose validity might vary across data sets. Therefore, other gene ranking validation methods should be further explored to assess the performance of a random forests-based gene ranking method and the EPS method in a wider range of RNA-Seq experiments. A gene set enrichment analysis performed on the genes highlighted by the two proposed methods showed that several cancer or cancer survival related pathways were significantly enriched, further supporting the claim that said methods yield genes associated with the biological context of each RNA-Seq dataset.

Replication experiments have shown that the gene rankings obtained with the two proposed methods varied across iterations. Given the way random forests operate, it should be noted that the variance in variable importance, which is used here to rank genes, decreases with increasing values of *n*_*tree*_. However, computational time also increases with *n*_*tree*_. There is thus a trade-off between variance reduction and method usability. It should also be noted that the EPS method seems to be de facto quasi-deterministic for some datasets, while having a high variance for others. A likely hypothesis for this behavior might be the greater differences in gene expression values between the 2 classes of samples in certain datasets vs. others.

Dataset size seems to have an effect on both the random-forest-based method and the EPS method. The nature of this effect however, can be traced back to not only the dataset size, but also the randomly selected samples. The EPS method uses the features of extreme samples on both sides of the linear separator. Choosing samples at random guarantees that the overall data properties will remain the same. Hence, the latent representation should not change drastically. However, decreasing the sample size lowers the chance of selecting extreme samples. This, in turn, dampens the ability to generate EPS further away from the linear separator. One should thus take sample size into consideration when selecting one or several gene selection methods in RNA-Seq experiments, as the supervised learning methods developed here perform best with larger sample sizes.

In conclusion, we have shown that using the permutation importance internally computed by the random forests algorithm, when said algorithm is used to build a classifier based on gene expression values of a case-control RNA-Seq data set, allowed to obtain a ranking of genes; Variational Autoencoders could be used to generate pseudo-samples mimicking the properties of real samples, albeit with extreme localizations in latent space; Using the feature weights of said pseudo-samples allowed to obtain a ranking of genes. These rankings were compared with the results of a differential expression analysis, with all three gene rankings being evaluated through survival analysis on a validation cohort different from the cohort used to generate both rankings. The results have shown that the random forests-based method and the EPS outperformed the differential expression-based method for 9 and 8 out of the 12 data sets analyzed, respectively. Although the genes selected by both methods are different, there is no significant difference in the number of highly correlated genes between both methods. Although the goal of this research is not to supersede differential expression analysis to select genes of interest in RNA-Seq studies, we have shown that differential expression analysis might miss out on important genes, and a supervised learning-based gene selection method should be used alongside.

As the field of machine learning contains many different supervised classification and feature selection algorithms, it would be of interest to extend this work by testing the performance of other methods for gene selection in the context of case-control RNA-Seq data sets.

## Author contributions

SW conceived and designed the experiments; performed the random forests analysis; contributed to the writing of the manuscript. RS developed and performed the Extreme-Pseudo Samples analysis; contributed to the writing of the manuscript.

### Conflict of interest statement

The authors declare that the research was conducted in the absence of any commercial or financial relationships that could be construed as a potential conflict of interest.

## Supplementary material

Supplementary Figure 1 Performance comparison of survival gene signatures. Evolution of the log-rank *p*-values obtained with survival gene signatures comprising incremental number of genes, for the 3 methods compared and the 8 smallest TCGA datasets.

Supplementary Table 1 Top 20 genes and related survival *p*-values obtained with DESeq2, RF, EPS, fold changes.

Supplementary Table 2 Gene rankings obtained with the 2 proposed methods.

Supplementary Table 3 Average correlation for the 50% most expressed genes, number of hierarchical clusters.

Supplementary Table 4 Average number of genes in common among the top 20 best ranked genes across 10 iterations for the two proposed methods.

Supplementary Table 5 Performance comparison of survival gene signatures obtained with fold-change magnitude-based gene selection. Color coding similar to Table 2.

Supplementary Table 6 Performance comparison of survival gene signatures obtained with a random selection of 20 percent of the initial samples. Color coding similar to Table 2.

## References

[B1] Anaissi A. Kennedy P. J. Goyal M. Catchpoole D. R. (2013). A balanced iterative random forest for gene selection from microarray data. BMC Bioinformatics 14:261. 10.1186/1471-2105-14-261 23981907 PMC3766035

[B2] Baserga R. Peruzzi F. Reiss K. (2003). The IGF-1 receptor in cancer biology. Int. J. Cancer 107, 873–877. 10.1002/ijc.11487 14601044

[B3] Brabender J. Danenberg K. D. Metzger R. Schneider P. M. Park J. Salonga D.. (2001). Epidermal growth factor receptor and HER2-neu mRNA expression in non-small cell lung cancer is correlated with survival. Clin. Cancer Res. 7, 1850–1855. 11448895

[B4] Bruchim I. Sarfstein R. Werner H. (2014). The IGF hormonal network in endometrial cancer: functions, regulation, and targeting approaches. Front. Endocrinol. 5:76. 10.3389/fendo.2014.00076 24904527 PMC4032924

[B5] Cao Y. Nimptsch K. Shui I. M. Platz E. A. Wu K. Pollak M. N.. (2015). Prediagnostic plasma IGFBP-1, IGF-1 and risk of prostate cancer. Int. J. Cancer 136, 2418–2426. 10.1002/ijc.29295 25348852 PMC4360136

[B6] Chou C. H. Yang N. K. Liu T. Y. Tai S. K. Hsu D. S. Chen Y. W.. (2012). Chromosome instability modulated by BMI1–AURKA signaling drives progression in head and neck cancer. Cancer Res. 73, 953–66. 10.1158/0008-5472.CAN-12-2397 23204235

[B7] Colaprico A. Silva T. C. Olsen C. Garofano L. Cava C. Garolini D.. (2015). TCGAbiolinks: an R/Bioconductor package for integrative analysis of TCGA data. Nucleic Acids Res. 44:e71. 10.1093/nar/gkv1507 26704973 PMC4856967

[B8] Dai C. Li N. Song G. Yang Y. Ning X. (2016). Insulin-like growth factor 1 regulates growth of endometrial carcinoma through PI3k signaling pathway in insulin-resistant type 2 diabetes. Am. J. Transl. Res. 8, 3329–3336. 27648123 PMC5009385

[B9] Danaee P. Ghaeini R. Hendrix D. A. (2017). A deep learning approach for cancer detection and relevant gene identification. Pac. Symp. Biocomput. 22, 219–229. 10.1142/9789813207813_0022 27896977 PMC5177447

[B10] Duro D. C. Franklin S. E. Dubé M. G. (2012). Multi-scale object-based image analysis and feature selection of multi-sensor earth observation imagery using random forests. Int. J. Remote Sens. 33, 4502–4526. 10.1080/01431161.2011.649864

[B11] Frères P. Wenric S. Boukerroucha M. Fasquelle C. Thiry J. Bovy N.. (2016). Circulating microRNA-based screening tool for breast cancer. Oncotarget 7, 5416–5428. 10.18632/oncotarget.6786 26734993 PMC4868695

[B12] Garber M. Grabherr M. G. Guttman M. Trapnell C. (2011). Computational methods for transcriptome annotation and quantification using RNA-seq. Nat. Methods 8, 469–477. 10.1038/nmeth.1613 21623353

[B13] Goel R. K. Lukong K. E. (2015). Tracing the footprints of the breast cancer oncogene BRK—past till present. Biochim. Biophys. Acta Rev. Cancer 1856, 39–54. 10.1016/j.bbcan.2015.05.001 25999240

[B14] Huang H. C. Niu Y. Qin L. X. (2015). Differential expression analysis for RNA-Seq: an overview of statistical methods and computational software: supplementary issue: sequencing platform modeling and analysis. Cancer Inform. 14, 57–67. 10.4137/CIN.S21631 26688660 PMC4678998

[B15] Joshi-Tope G. Gillespie M. Vastrik I. D'Eustachio P. Schmidt E. de Bono B.. (2005). Reactome: a knowledgebase of biological pathways. Nucleic Acids Res. 33, D428–D432. 10.1093/nar/gki072 15608231 PMC540026

[B16] Junk D. J. Bryson B. L. Smigiel J. M. Parameswaran N. Bartel C. A. Jackson M. W. (2017). Oncostatin M promotes cancer cell plasticity through cooperative STAT3-SMAD3 signaling. Oncogene 36, 4001–4013. 10.1038/onc.2017.33 28288136 PMC5509502

[B17] Kamburov A. Stelzl U. Lehrach H. Herwig R. (2012). The ConsensusPathDB interaction database: 2013 update. Nucleic Acids Res. 41, D793–D800. 10.1093/nar/gks1055 23143270 PMC3531102

[B18] Kanehisa M. Goto S. (2000). KEGG: kyoto encyclopedia of genes and genomes. Nucleic Acids Res. 28, 27–30. 10.1093/nar/28.1.27 10592173 PMC102409

[B19] Kingma D. P. Welling M. (2013). Auto-encoding variational bayes. arXiv:1312.6114[preprint].

[B20] Love M. I. Huber W. Anders S. (2014). Moderated estimation of fold change and dispersion for RNA-seq data with DESeq2. Genome Biol. 15:550. 10.1186/s13059-014-0550-8 25516281 PMC4302049

[B21] Michalik L. Desvergne B. Wahli W. (2004). Peroxisome-proliferator-activated receptors and cancers: complex stories. Nat. Rev. Cancer 4, 61–70. 10.1038/nrc1254 14708026

[B22] Mortazavi A. Williams B. A. McCue K. Schaeffer L. Wold B. (2008). Mapping and quantifying mammalian transcriptomes by RNA-Seq. Nat. Methods 5, 621–628. 10.1038/nmeth.1226 18516045 PMC13303166

[B23] Muto J. Shirabe K. Sugimachi K. Maehara Y. (2015). Review of angiogenesis in hepatocellular carcinoma. Hepatol. Res. 45, 1–9. 10.1111/hepr.12310 24533487

[B24] Phillips P. C. (2008). Epistasis—the essential role of gene interactions in the structure and evolution of genetic systems. Nat. Rev. Genet. 9, 855–867. 10.1038/nrg2452 18852697 PMC2689140

[B25] Sanna C. Rosso C. Marietti M. Bugianesi E. (2016). Non-alcoholic fatty liver disease and extra-hepatic cancers. Int. J. Mol. Sci. 17:E717. 10.3390/ijms17050717 27187365 PMC4881539

[B26] Schrider D. R. Kern A. D. (2018). Supervised machine learning for population genetics: a new paradigm. Trends Genet. 34, 301–312. 10.1016/j.tig.2017.12.005 29331490 PMC5905713

[B27] Tan J. Ung M. Cheng C. Greene C. S. (2014). Unsupervised feature construction and knowledge extraction from genome-wide assays of breast cancer with denoising autoencoders. Pac. Symp. Biocomput. 14, 132–143. 10.1142/9789814644730_0014 25592575 PMC4299935

[B28] Trapnell C. Pachter L. Salzberg S. L. (2009). TopHat: discovering splice junctions with RNA-Seq. Bioinformatics 25, 1105–1111. 10.1093/bioinformatics/btp120 19289445 PMC2672628

[B29] Vidal M. Cusick M. E. Barabási A. L. (2011). Interactome networks and human disease. Cell 144, 986–998. 10.1016/j.cell.2011.02.016 21414488 PMC3102045

[B30] Vogelstein B. Papadopoulos N. Velculescu V. E. Zhou S. Diaz L. A. Kinzler K. W. (2013). Cancer genome landscapes. Science 339, 1546–1558. 10.1126/science.1235122 23539594 PMC3749880

[B31] Wang Z. Gerstein M. Snyder M. (2009). RNA-Seq: a revolutionary tool for transcriptomics. Nat. Rev. Genet. 10, 57–63. 10.1038/nrg2484 19015660 PMC2949280

[B32] Weinstein J. N. Collisson E. A. Mills G. B. Shaw K. R. M. Ozenberger B. A. Ellrott K.. (2013). The cancer genome atlas pan-cancer analysis project. Nat. Genet. 45, 1113–1120. 10.1038/ng.2764 24071849 PMC3919969

[B33] Wen Y. A. Xing X. Harris J. W. Zaytseva Y. Y. Mitov M. I. Napier D. L.. (2017). Adipocytes activate mitochondrial fatty acid oxidation and autophagy to promote tumor growth in colon cancer. Cell Death Dis. 8:e2593. 10.1038/cddis.2017.21 28151470 PMC5386470

[B34] Wenric S. ElGuendi S. Caberg J. H. Bezzaou W. Fasquelle C. Charloteaux B.. (2017). Transcriptome-wide analysis of natural antisense transcripts shows their potential role in breast cancer. Sci. Rep. 7:17452. 10.1038/s41598-017-17811-2 29234122 PMC5727077

[B35] Wright M. N. Ziegler A. (2015). Ranger: a fast implementation of random forests for high dimensional data in C++ and R. arXiv:1508.04409[preprint].

[B36] Yao D. Yang J. Zhan X. Zhan X. Xie Z. (2015). A novel random forests-based feature selection method for microarray expression data analysis. Int. J. Data Min. Bioinform. 13, 84–101. 10.1504/IJDMB.2015.070852 26529910

[B37] Yates L. R. Campbell P. J. (2012). Evolution of the cancer genome. Nat. Rev. Genet. 13, 795–806. 10.1038/nrg3317 23044827 PMC3666082

[B38] Zhu M. Che Q. Liao Y. Wang H. Wang J. Chen Z.. (2015). Oncostatin M activates STAT3 to promote endometrial cancer invasion and angiogenesis. Oncol. Rep. 34, 129–138. 10.3892/or.2015.3951 25954856

