# Supplementary figures and images for: Using Supervised Learning Methods for Gene Selection in RNA-Seq Case-Control Studies
Source: Front Genet. 2018 Aug 3;9:297. doi: 10.3389/fgene.2018.00297 (PMC6085558; doi:10.3389/fgene.2018.00297)

TCGA-HNSC

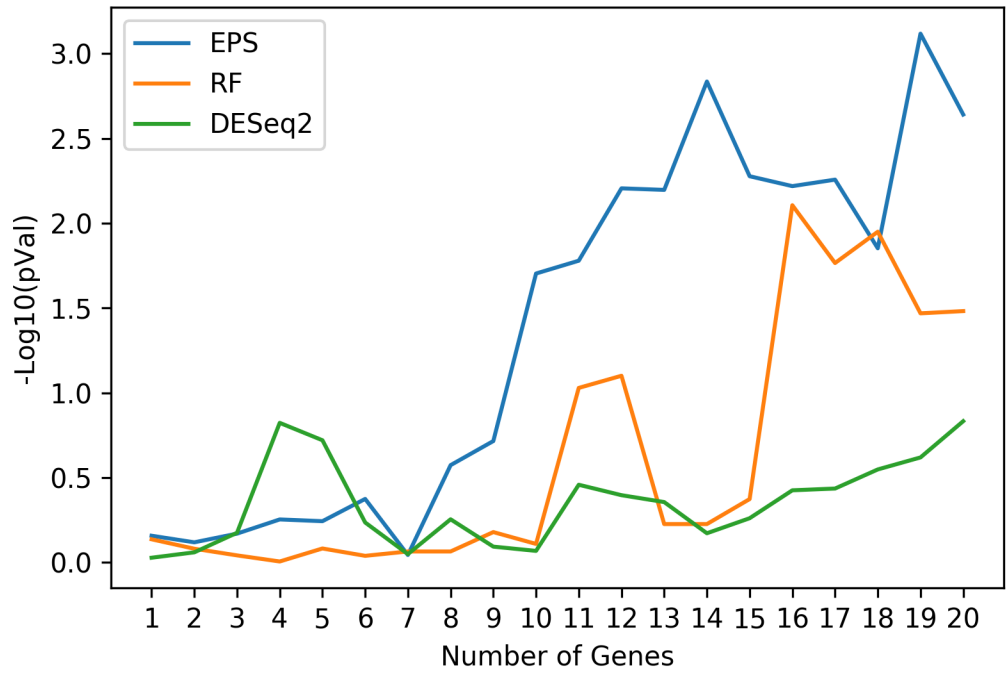

TCGA-THCA

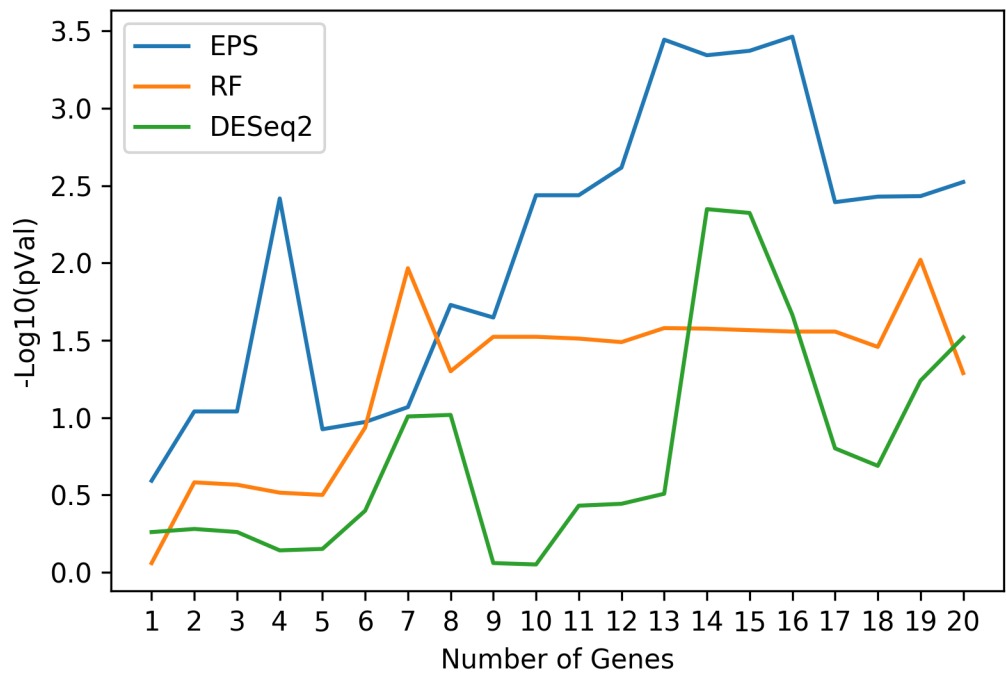

TCGA-LUSC

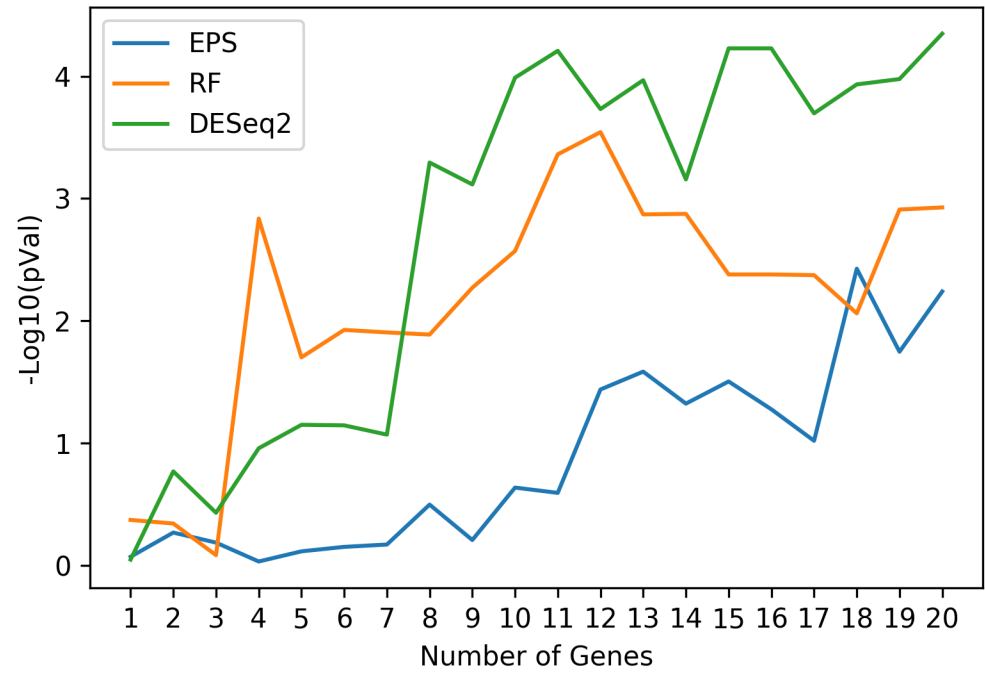

TCGA-PRAD

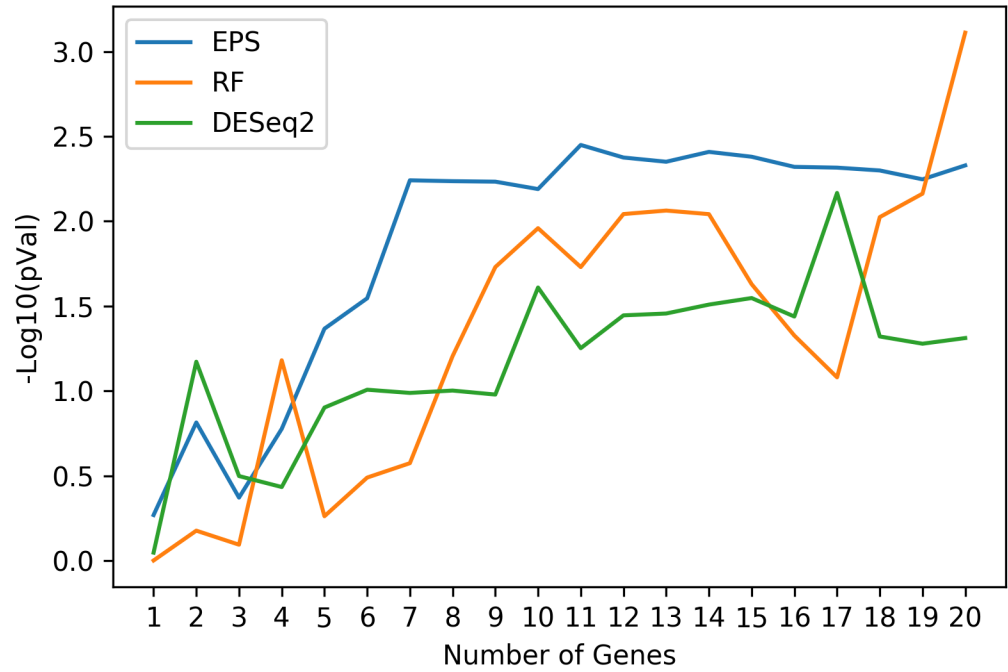

TCGA-COAD

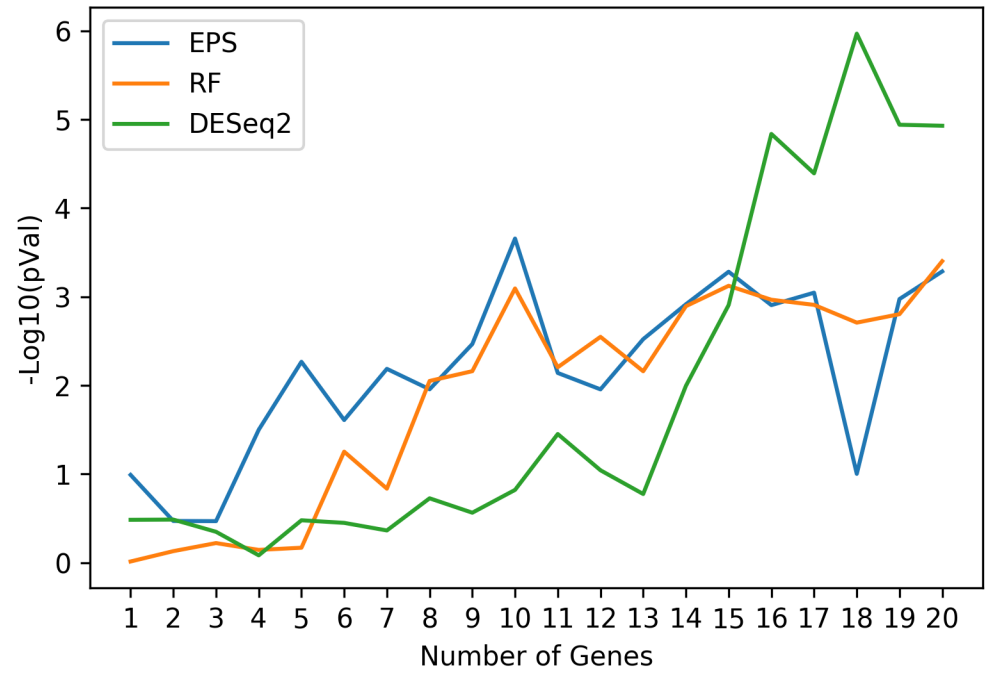

TCGA-STAD

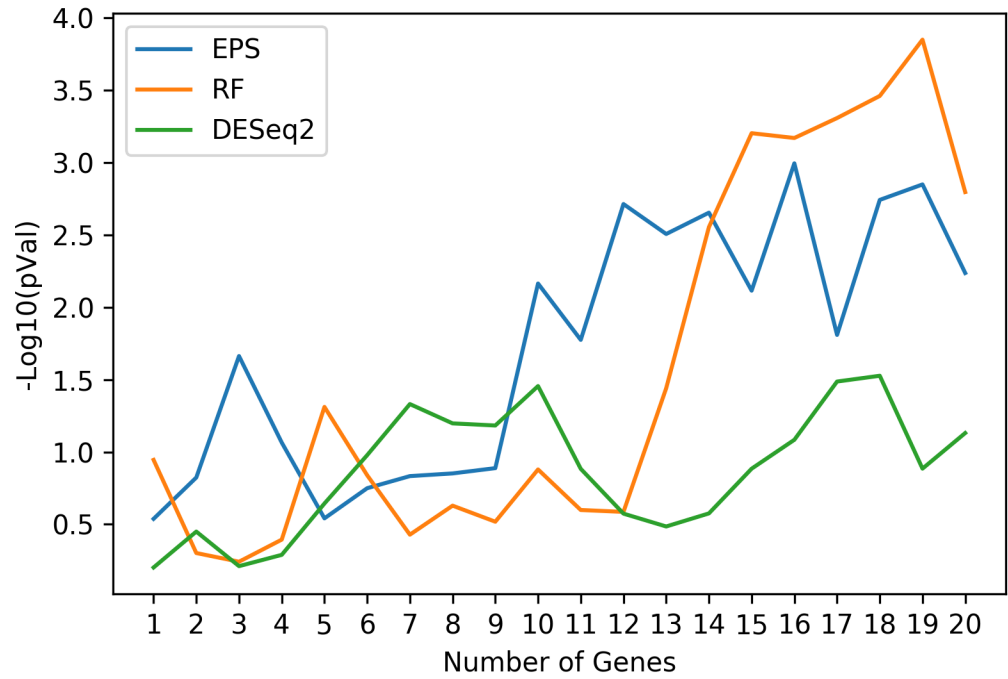

TCGA-LIHC

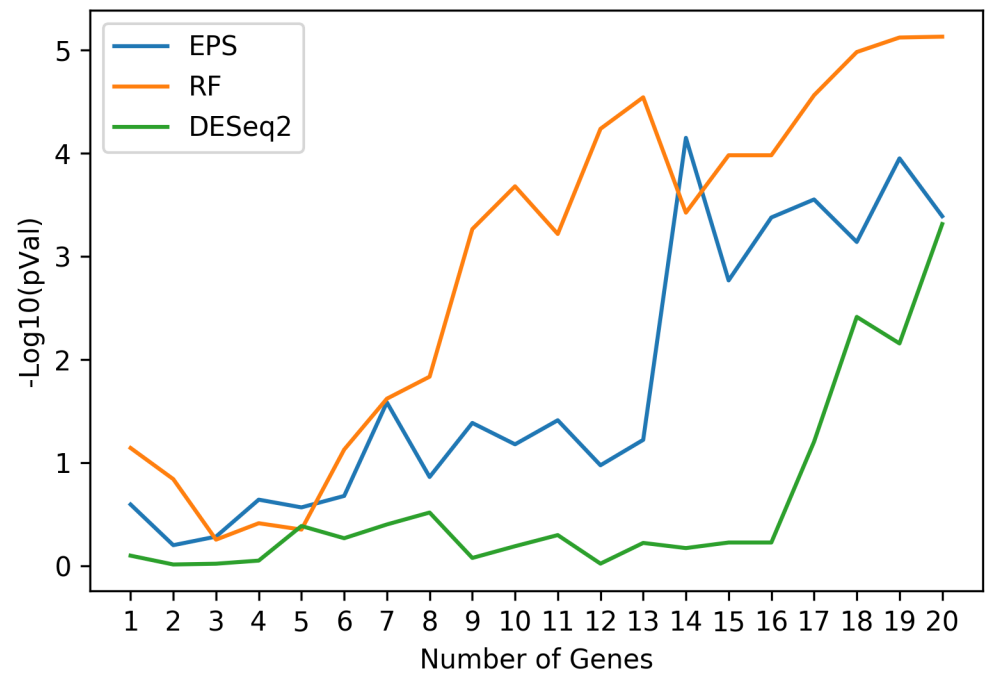

TCGA-KIRP

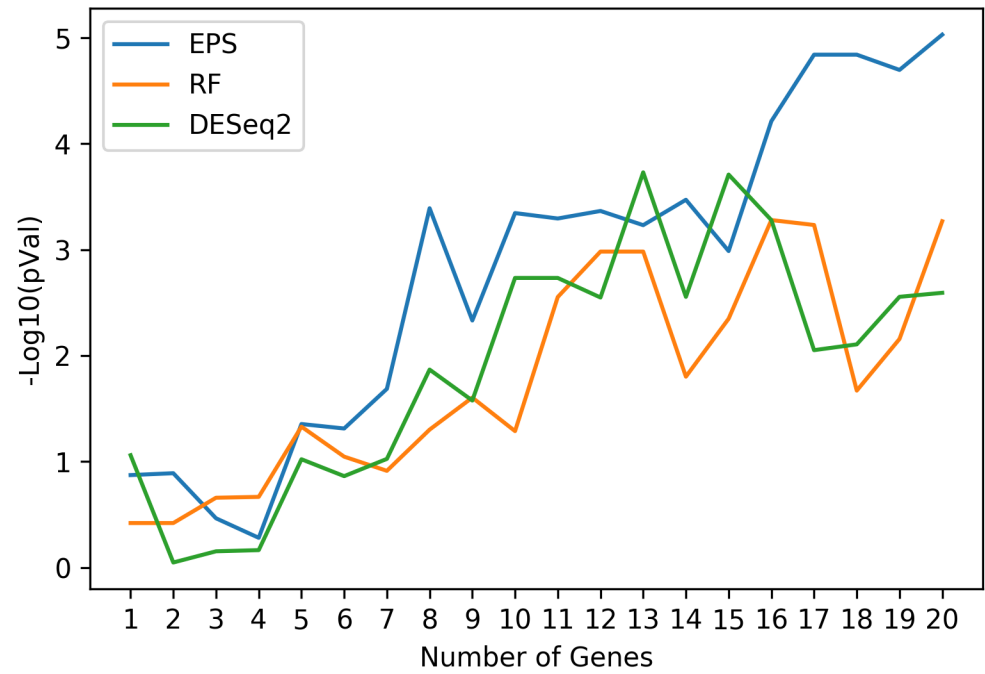

Supplement: Supplementary Figure 1 — Performance comparison of survival gene signatures. Evolution of the log-rank p-values obtained with survival gene signatures comprising incremental number of genes, for the 3 methods compared and the 8 smallest TCGA datasets. [file Image_1.PDF]
